# Leptin Activates Oxytocin Neurons of the Hypothalamic Paraventricular Nucleus in Both Control and Diet-Induced Obese Rodents

**DOI:** 10.1371/journal.pone.0059625

**Published:** 2013-03-18

**Authors:** Mario Perello, Jesica Raingo

**Affiliations:** 1 Laboratory of Neurophysiology, Argentine Research Council and Scientific Research Commission of the Province of Buenos Aires, La Plata, Buenos Aires, Argentina; 2 Laboratory of Electrophysiology of the Multidisciplinary Institute of Cell Biology, Argentine Research Council and Scientific Research Commission of the Province of Buenos Aires, La Plata, Buenos Aires, Argentina; University of Michigan, United States of America

## Abstract

The adipocyte-derived hormone leptin acts in the brain to reduce body weight and fat mass. Recent studies suggest that parvocellular oxytocin (OXT) neurons of the hypothalamic paraventricular nucleus (PVN) can mediate body weight reduction through inhibition of food intake and increased energy expenditure. However, the role of OXT neurons of the PVN as a primary target of leptin has not been investigated. Here, we studied the potential role of OXT neurons of the PVN in leptin-mediated effects on body weight regulation in fasted rats. We demonstrated that intracerebroventricular (ICV) leptin activates STAT3 phosphorylation in OXT neurons of the PVN, showed that this occurs in a subpopulation of OXT neurons that innervate the nucleus of the solitary tract (NTS), and provided further evidence suggesting a role of OXT to mediate leptin’s actions on body weight. In addition, our results indicated that OXT neurons are responsive to ICV leptin and mediate leptin effects on body weight in diet induced obese (DIO) rats, which are resistant to the anorectic effects of the hormone. Thus, we conclude that leptin targets a specific subpopulation of parvocellular OXT neurons of the PVN, and that this action may be important for leptin’s ability to reduce body weight in both control and obese rats.

## Introduction

The adipocyte-derived hormone leptin regulates key behavioral, autonomic and neuroendocrine responses necessary to maintain body energy balance [1]. Leptin acts in the brain to reduce body weight and fat mass through inhibition of food intake and increased energy expenditure [1]. Leptin actions are mediated by the leptin receptor (LepR), which is expressed in several brain regions [2], [3]. Among them, the hypothalamic arcuate nucleus (ARC) has become major focus in the leptin-mediated regulation of energy balance field [4]. In contrast, leptin action on other hypothalamic regions has received less attention. The PVN is another key site for maintaining energy balance [5]. We, and others, have contributed to show that leptin can directly activate some PVN neurons, such the hypophysiotropic thyrotropin releasing hormone (TRH) neurons that regulate the hypothalamic-pituitary-thyroid axis [6]–[9]. However, other leptin targets within the PVN are not well established. Recent studies suggest that parvocellular OXT neurons of the PVN are able to inhibit food intake and increase energy expenditure [10]–[16]. Despite that OXT neurons express LepR [17], the potential role of these neurons as a primary target of leptin has not been investigated. Thus, an aim of this study was to test the hypothesis that leptin activates OXT neurons of the PVN and that this action affects body weight.

A better understanding of the neural circuits by which leptin regulate body weight is essential to develop new strategies for the treatment of obesity. Most obese people exhibit elevated circulating leptin levels that fail to suppress feeding or prevent body weight gain [1]. Although a number of mechanisms have been proposed to explain this leptin resistance, the molecular reasons that cause this state are still under debate [18]. A classical animal model of obesity is DIO rodents, which are unresponsive to the anorectic effect of leptin [19], [20]. Interestingly, the decrease in leptin signaling observed in DIO models specifically occurs in the ARC but not in other leptin-responsive sites of the hypothalamus [21]–[23]. For instance, we have shown that TRH neurons of the PVN in DIO animals remain sensitive to the direct action of leptin and that the hyperleptinemic state of obese animals affects the set point of the hypothalamic-pituitary-thyroid axis [23]. In addition, a recent study by Enriori *et al.* showed that leptin activates hypothalamic dorsomedial nucleus in DIO rodents causing sympathetic activation and increase of brown adipose tissue (BAT) temperature [24]. Whether additional hypothalamic neuronal populations remain sensitive to leptin in DIO animals is currently unknown. Thus, we also tested the hypothesis that OXT neurons of the PVN remain sensitive to leptin in DIO animals.

## Materials and Methods

### Animals and Stereotaxic Surgeries

Male Sprague-Dawley rats were generated in the animal facility of the IMBICE. At 22 days of age, rats were fed a regular (3.3 kcal/g of energy, 12.1% fat) or high fat rodent (5.24 kcal/g of energy, 60.0% fat) diets, as done in the past [23]. Food and water were available *ad libitum* unless otherwise indicated. For the studies, rats fed on regular or high fat diet for 12 weeks were stereotaxically implanted with an ICV 21-gauge guide cannula (Plastic One), as previously detailed [23]. The placement coordinates for the lateral ventricle were: AP −0.8 mm to bregma, L −.2 mm and V −3.6 mm. After surgery, ICV-cannulated rats were caged individually and allowed to recover for 6–7 days. Correct placement of the cannulas was verified by measurement of water intake in response to ICV Angiotensin II [23]. This study was carried out in strict accordance with the recommendations in the Guide for the Care and Use of Laboratory Animals of the National Institutes of Health, and all efforts were made to minimize suffering. The protocol was approved by the Institutional Animal Care and Use Committee of the Multidisciplinary Institute of Cell Biology (approval ID 10-0113).

### Tracing Studies

The retrograde tracer fluoro-gold (FG, Molecular Probes) was used to trace PVN projections. To label PVN neurons sending terminals to the posterior pituitary gland, ICV-cannulated rats were injected intraperitoneally with FG (15 mg/kg body weight of 2.5% solution in saline) 48 h before perfusion [23]. To label neurons sending terminals to the NTS, FG was stereotaxically microinjected bilaterally into this nucleus. The placement coordinates were: AP −4.3 mm to interaural line, L 0.9 mm and V −7.1 mm. Our goal was to hit mainly the medial and gelatinosus subdivisions of the NTS, which received the densest OXT axon innervations [25]. These coordinates were initially obtained from the Paxinos and Watson atlas [26] and then modified based on analysis of the injection sites in pilot studies. A 0.4 µL volume of 0.2% FG in saline was injected via a 33-gauge injector. Same day, rats were implanted with an ICV cannula. We then waited 6 days for retrograde transport of the FG before starting the experiment.

### Experimental Designs

#### 1- ICV leptin treatment in fasted rats and neuroanatomical studies

ICV-cannulated rats were fasted for 48 h, beginning at 12∶00 p.m. Between 12∶00 and 1∶00 p.m. on the third day, fasted rats ICV injected given with vehicle (artificial cerebrospinal fluid [23]) alone or containing recombinant murine leptin (3.5 µg/rat, obtained from Dr. E. Parlow of The National Hormone and Pituitary Program). All ICV injections were made in freely moving rats through a 30 gauge needle that extends 0.5 mm below the guide cannula. After 30 min, rats were anesthetized and systemically perfused with 4% paraformaldehyde. Blood samples were taken to measure plasma leptin levels using commercial RIA kits (Linco Research). Brains were then removed, post-fixed and cryoprotected in 20% sucrose solution. Brains were frozen, cut in 3 series of 25-µm-thick coronal sections, which were used for immunohistochemistry. Neuroanatomical studies were performed in vehicle- and leptin-treated control (n = 3 and 5, respectively) and DIO (n = 3 and 5, respectively) rats. Also, we used ICV leptin injections in control rats that had FG injections performed either systemically (n = 5) or in the NTS (n = 7). Of note, stereotaxic microinjections of FG in the NTS resulted in 3 missed injections centered outside of either the medial or gelatinosus subdivisions; these rats were excluded of the analysis.

#### 2- ICV leptin treatment in fasted rats and gene expression analysis

ICV-cannulated control and DIO rats were divided into three groups fed, fasted and fasted plus leptin. The fed group was allowed free access to food and ICV-injected with vehicle (n = 5 and 6 for control and DIO group, respectively). The second group was fasted for 48 h, beginning at 12∶00 p.m. and ending between 12∶00 and 1∶00 p.m. on the third day, and ICV-injected with vehicle, as described below (n = 13 and 7 for control and DIO groups, respectively). Fasted plus leptin group was performed using two different protocols. In one set of fasted rats, leptin (3.5 µg/rat) was administered ICV once between 12∶00 and 12∶30 p.m. on the third day of fasting, and rats were sacrificed 2 h later (n = 5). In other set of fasted rats, leptin (3.5 µg/rat) was ICV administered every 6 h, beginning 6 h after the removal of food and ending 6 h after completion of 48 h of fasting (n = 7 and 6 for control and DIO groups, respectively). Rats were weighed and sacrificed by decapitation around 12∶00 p.m. on the third day of fasting. The PVN-enriched sections (hereafter referred to as PVN) were dissected out for mRNA quantifications. Brains were dissected and sectioned into 1 mm coronal slices as described in the past [27], [28]. Micro-dissections of tissue corresponding to the location of the PVN, identified by comparing the coronal slices to a rat brain atlas of Paxinos and Watson [26], were excised using a 15 g needle. The PVN punches sections were collected in TRIzol Reagent (Invitrogen) for RNA isolation.

#### 3- OXT blockage in ICV vehicle- and leptin-treated fasted rats

ICV-cannulated control and DIO rats were fasted for 48 h, beginning at 12∶00 p.m and ending at 12∶00 p.m on the third day, and ICV-injected with vehicle containing an OXT receptor antagonist [25 ng/rat (β-mercapto-β,β cyclopentamethylenepropionyl^1^, O-Me-Tyr^2^, Orn^8^)-OXT; Sigma Aldrich] alone or plus leptin (3.5 µg/rat). ICV administrations were performed every 6 h, beginning 6 h after the removal of food and ending 6 h after completion of 48 h of fasting, when body weight of rats was determined. All experimental groups had 6 rats.

### Immunohistochemistry (IHC)

Immunostaining was performed as described before [7]. Briefly, coronal section were sequentially treated with 1% H_2_O_2_, 0.03% SDS and 4% normal donkey serum and then incubated with rabbit anti-phosphorylated signal transducer and activator of transcription 3 (pSTAT3) antibody [1∶1500, Cell Signaling, cat. #9145] overnight at 4°C. Next day, sections were incubated with biotinylated donkey anti-rabbit antibody (1∶1,000), followed by avidin-biotin complex solution and brown precipitate development by diaminobenzidine solution. Then, brain slices were incubated overnight at 4°C with goat anti-Neurophysin I (NPI) antibody (1∶5,000, Santa Cruz, cat sc-7810). NPI is a protein synthesized from the same OXT precursor extensively validated and used to identify OXT-producing neurons [29]. Next day, sections were incubated with red fluorescent donkey anti-goat Alexa 594 antibody (Molecular Probes), mounted and cover slipped in a fluorescence mounting solution. In tracing studies, triple pSTAT3/NPI/FG immunostaining was performed in order to increase the FG fluorescent signal. FG staining was performed by overnight incubation with guinea pig anti-FG antibody (1∶3,000, Protos Biotech) at 4°C, and then visualization was done with donkey anti-guinea pig Alexa 488 antibody (Molecular Probes). Results were visualized using either fluorescence (NPI and FG) or bright-field light (pSTAT3) sources. Images were acquired with a Nikon E800 microscope and a Spot II digital camera (Diagnostic Instruments). Adobe Photoshop CS2 software was used to adjust levels, contrast and brightness and to combine fluorescence and bright-field images.

### Quantitative Neuroanatomical Analysis

Double-labeled and triple-labeled sections were used for quantitative analysis. Total NPI-immunoreactive (IR) neurons and NPI-IR neurons with nuclei positive for pSTAT3 were counted on each side of the third ventricle. The relationship was expressed as a percentage, which represents NPI-IR cells positive for pSTAT3 compared to the total number of NPI-IR cells observed [8]. Quantifications were performed in complete series of coronal sections and then numbers were multiply by three. Data were corrected for double counting, according to the method of Abercrombie [30], where the ratio of the actual number of neurons to the observed number is represented by T/(T+h) where T = section thickness, and h = the mean diameter of the neuron. For this, average NPI-IR cell diameter, in at least 40 cells per PVN, was quantified using NIH image software Image J in each experimental group. In the case of triple staining, we quantified all double labeled FG/NPI-IR, single labeled FG-IR (which were negative for NPI) and single labeled NPI-IR (which were negative for FG) neurons of the PVN and then calculate the percentage of them that were positive for pSTAT3.

### Gene Expression Analysis

Total RNA was isolated using TRIzol Reagent and quantified by absorbance at 260 nm. Total RNA was treated with DNase I (Roche). cDNA synthesis was generated using random hexamer primers and SuperScript III reverse transcriptase (Invitrogen). Quantitative real-time PCRs were done using the SYBR Green® PCR Core Reagents and the ABI 7500 Fast-Real time PCR system (Applied Biosystems). Averaged mRNA levels of *OXT* were normalized to the housekeeping gene hypoxanthine phosphoribosyl transferase (hprt1), calculated by the comparative threshold cycle (Ct) method [31], and presented as relative of levels observed in PVN of control fed rats. Primers sequences were: upstream *OXT*, 5′-TGCCCCAGTCTCGCTTGCT-3′; downstream *OXT*, 5′-TCCAGGTCTAGCGCAGCCC-3′; upstream *hprt1*, 5′-GCAGACTTTGCTTTCCTTGG-3′; downstream *hprt1*, 5′-GTCTGGCCTGTATCCAACACT-3′. All reactions were performed per triplicate. Standard curves for *OXT* and *hprt* 1 transcript levels were generated using hypothalamic cDNA of control fed rat.

### Statistical Analyses

Data is expressed as the mean±SEM. The t-test was used to compare percentage NPI-IR neurons positive for pSTAT3 of the PVN in vehicle- vs. leptin-treated groups. Also, t-test was performed when comparing data of control vs. DIO groups or vehicle vs. leptin effects on the different experimental paradigms. One-way ANOVA was performed when comparing OXT mRNA levels. Two-way ANOVA, with leptin and OXT receptor antagonist as factors, was performed when comparing body weight data. Significant differences were considered when p<0.05.

## Results

### Leptin Activates OXT Neurons of the PVN

**Figure 1:**
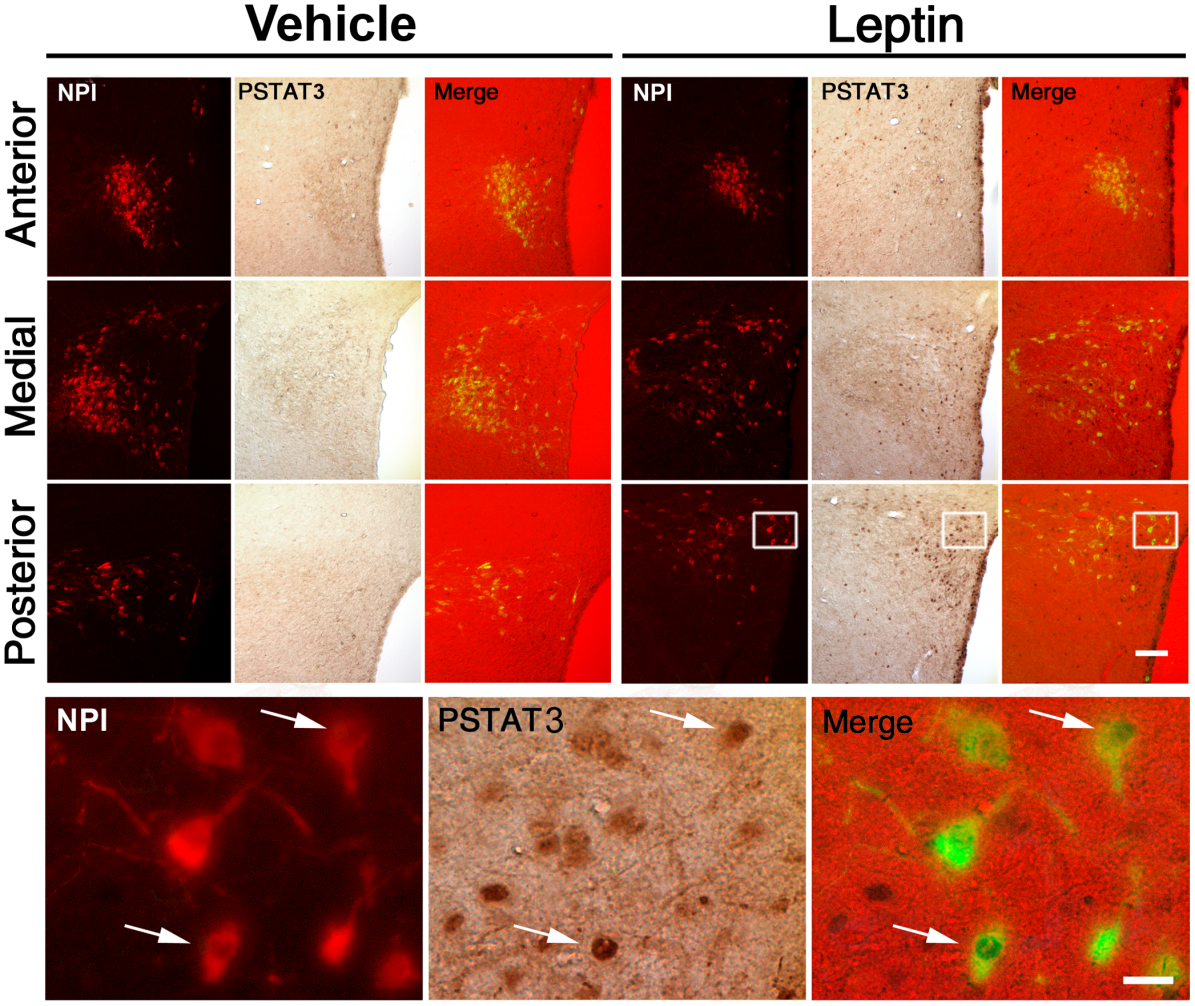
ICV leptin increased pSTAT3 in OXT neurons of the PVN in fasted control rats. Left and right upper sets of panels depict low magnification photomicrographs of the anterior, medial and posterior parts of the PVN in fasted control rats treated with vehicle or leptin, respectively. Red fluorescent and brown signals label NPI-IR and pSTAT3-IR cells, respectively. Double pSTAT3/NPI-IR cells are shown in the merge panels. Bottom set of panels show high magnification photomicrographs of area marked in low magnification images; arrows point to dual-labeled cells. Scale bars: 50 µm (Upper panels), 20 µm (Bottom panels).

To test if OXT neurons respond to leptin, we performed double IHC for pSTAT3 and NPI, as a marker of OXT producing neurons, in leptin- and vehicle-treated fasted rats. Red NPI signal was confined to the perikarya and dendrites. FG signal was seen as cytoplasmic granular green staining. PSTAT3 signal was seen as nuclear brown labeling. Figure 1 depicts a set of representative images of three levels of the PVN of rats subjected to vehicle or leptin treatments. NPI-IR cells were concentrated in the magnocellular division of the PVN but also scattered found within all five parts of the parvocellular division of the PVN, as previously described [32]. Total number of NPI-IR cells of the PVN was not significantly affected by leptin (1381±203 and 1467±219 cells in vehicle- and leptin-groups, respectively). We found no NPI-IR neurons positive for pSTAT3 in vehicle-treated rats, while 6.2±2.1% of NPI-IR neurons of the PVN were positive for pSTAT3 in leptin-treated rats (p<0.01 vs. vehicle-treated rats). Thus, leptin is able to act on some OXT neurons of the PVN.

### OXT Neurons Responsive to Leptin Innervate the NTS

**Figure 2:**
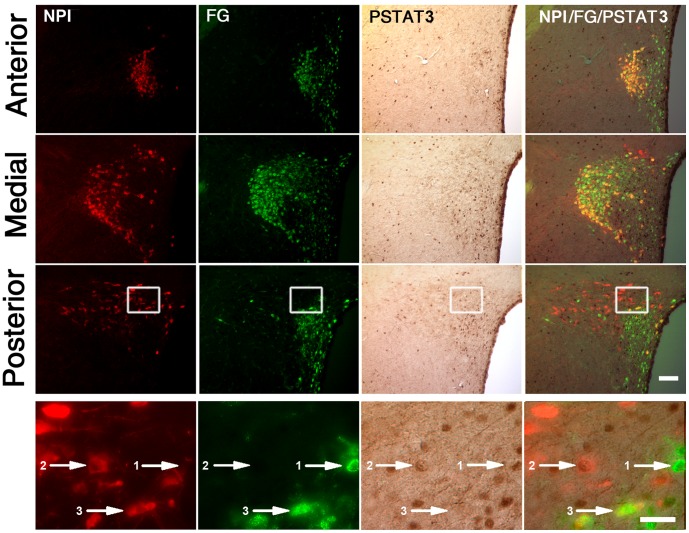
ICV leptin failed to increase pSTAT3 in OXT neurons of the PVN belonging to the hypophyseal system. Upper sets of panels depict low magnification photomicrographs of the anterior, medial and posterior parts of the PVN of fasted rats subjected to intraperitoneal FG injection, ICV leptin treatment and further triple NPI, FG and pSTAT3 staining. Right column of images shows merge of NPI (red fluorescent staining), FG (green fluorescent staining) and pSTAT3 (brown staining) signals. Bottom set of panels show high magnification photomicrographs of area marked in low magnification images. Numbered arrows point to dual-labeled cells as follows: 1-double pSTAT3/FG-IR cell, negative for NPI; 2-double pSTAT3/NPI-IR cell, negative for FG; 3-double FG/NPI-IR cell, negative for pSTAT3. Scale bars: 50 µm (Upper panels), 20 µm (Bottom panels).

Initially, we tested if leptin-responsive OXT neurons send their axons terminals outside of the blood-brain barrier [33]. Figure 2 depicts a set of representative images of the PVN of fasted rats subjected to intraperitoneal FG injection, ICV leptin treatment and further triple NPI, FG and pSTAT3 staining. Double FG/NPI-IR cells were located in the anterior, medial and posterior parts of the magnocellular division of the PVN and estimated in 968±163 cells. All double FG/NPI-IR neurons of the PVN were negative for pSTAT3. Single NPI-IR cells were found scattered within all parts of the parvocellular division of the PVN and estimated in 419±76 cells. Quantitative analysis indicated that 18±5% of these single NPI-IR cells were positive for pSTAT3. Of note, a fraction (∼21%) of single FG-IR neurons of the PVN was positive for pSTAT3. Thus, leptin-responsive OXT neurons do not belong to the hypophyseal system, and a fraction of the leptin-responsive non-OXT neurons does project to the pituitary gland.

**Figure 3:**
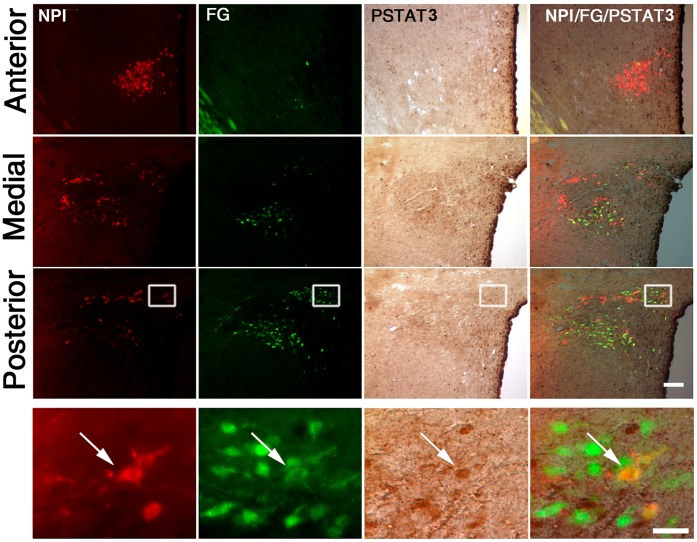
ICV leptin increased pSTAT3 in OXT neurons of the PVN projecting to the NTS. Upper sets of panels depict low magnification photomicrographs of the anterior, medial and posterior parts of the PVN of rats with stereotaxic injections of FG in the NTS and ICV treated with leptin. Right column of images shows merge of NPI (red fluorescent staining), FG (green fluorescent staining) and pSTAT3 (brown staining) signals. Bottom set of panels show high magnification photomicrographs of area marked in low magnification images, with arrow pointing to a triple-labeled cell. Scale bars: 50 µm (Upper panels), 20 µm (Bottom panels).

OXT parvocellular neurons of the PVN project to specific autonomic preganglionic neurons located in the intermedio-lateral cell column of the spinal cord and in the NTS [32]. However, leptin-activated neurons of the PVN do not innervate pre-ganglionic neurons in the spinal cord [34]. Thus, we tested if leptin-responsive OXT neurons send their projections to the NTS. Figure 3 depicts a set of representative images of the PVN of fasted rats subjected to FG injection in the NTS, ICV leptin treatment and further triple NPI, FG and pSTAT3 staining. As previously reported, FG-IR cells were scattered mostly within the dorsal, lateral and medial parvocellular regions of the PVN [32]. No double FG/NPI-IR cells were observed outside the PVN. Double FG/NPI-IR cells were concentrated in the caudal part of the parvocellular division of the PVN and estimated in 108±16 cells. Quantitative analysis indicated that 65.7±8.9% of double FG/NPI-IR neurons of the PVN were positive for pSTAT3. Interestingly, a fraction (∼19%) of single FG-IR neurons of the PVN was positive for pSTAT3. Of note, all single NPI-IR neurons were negative for pSTAT3. Thus, our data suggest that leptin exclusively activates OXT neurons of the parvocellular PVN that innervate the NTS.

### Leptin Affects OXT Gene Expression in the PVN

**Figure 4:**
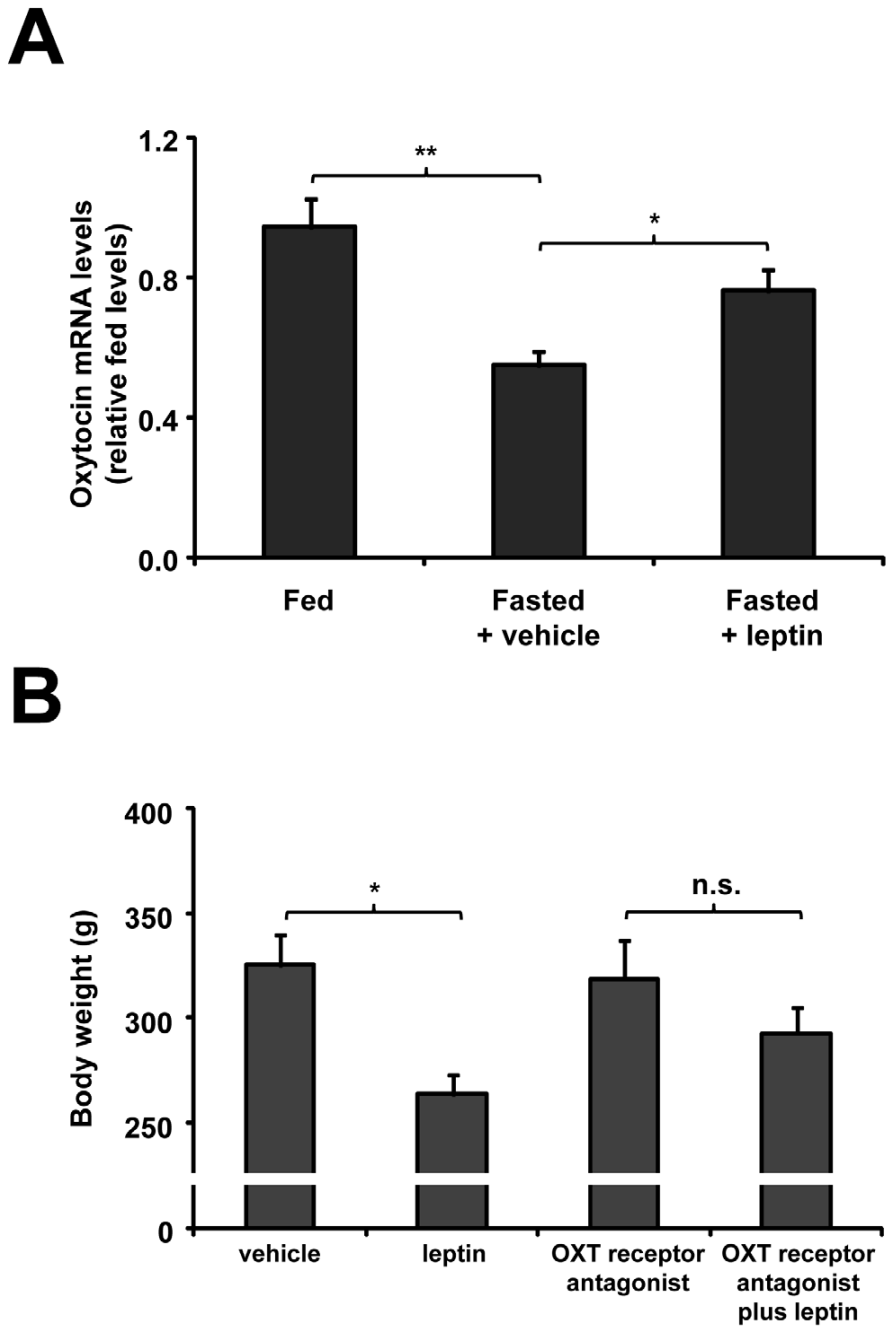
ICV leptin affected OXT gene expression in the PVN (A) and required OXT signaling to regulate body weight in fasted control rats (B). **A.** OXT mRNA levels of *ad libitum* fed, fasted and leptin-treated fasted rats are presented as relative of levels observed in PVN of control fed rats. **B.** Body weight was determined after completion of 48 h fasting and simultaneous ICV administrations, every 6 h, of either vehicle or leptin in presence or absence of the OXT receptor antagonist. *, p<0.05. **, p<0.01. n.s., not significant.

We next determined if leptin action on OXT neurons affects the neuropeptide gene expression. Plasma leptin levels were significantly decreased in control fasted rats (2.1±0.5 vs. 6.2±1.3 ng/mL in fed condition). As shown in Figure 4A, OXT mRNA levels in the PVN significantly decreased in fasted rats as compared to fed group (0.55±0.06 vs. 1.00±0.06 relative levels, p<0.01). Two hour after ICV leptin treatment, OXT mRNA levels in the PVN were unchanged (0.59±0.05 relative levels). Thus, we used a different protocol in which rats were ICV treated with leptin every 6 h during the fasting period. Using this protocol, we found that leptin significantly increased OXT mRNA levels in the PVN (0.76±0.07 relative levels, p<0.01 as compared to fasted rats treated with vehicle). As shown in Figure 4B, fasted rats receiving ICV leptin showed a further reduction of body weight, as compared to fasted rats treated with vehicle (264±10 vs. 325±15 g, respectively, p<0.01). Thus, our findings suggest that ICV leptin increases OXT mRNA levels in the PVN of fasted rats.

### Leptin Requires OXT Signaling to Regulate Body Weight in Fasted Rats

Then, we tested the effect of an OXT receptor antagonist on leptin-induced reduction of body weight. ICV treatment of fasted rats with the OXT receptor antagonist alone did not affect body weight (319±18 g), as compared to fasted rats treated with vehicle. Fasted rats receiving leptin and OXT receptor antagonist had a body weight of 293±12 g, which was not significantly different of the body weight of fasted rats receiving an OXT receptor antagonist alone (Figure 4B). Thus, OXT signaling blockage attenuates the leptin-induced reduction of body weight in fasted rats.

### Leptin Activates OXT Neurons of the PVN in DIO Rats

**Figure 5:**
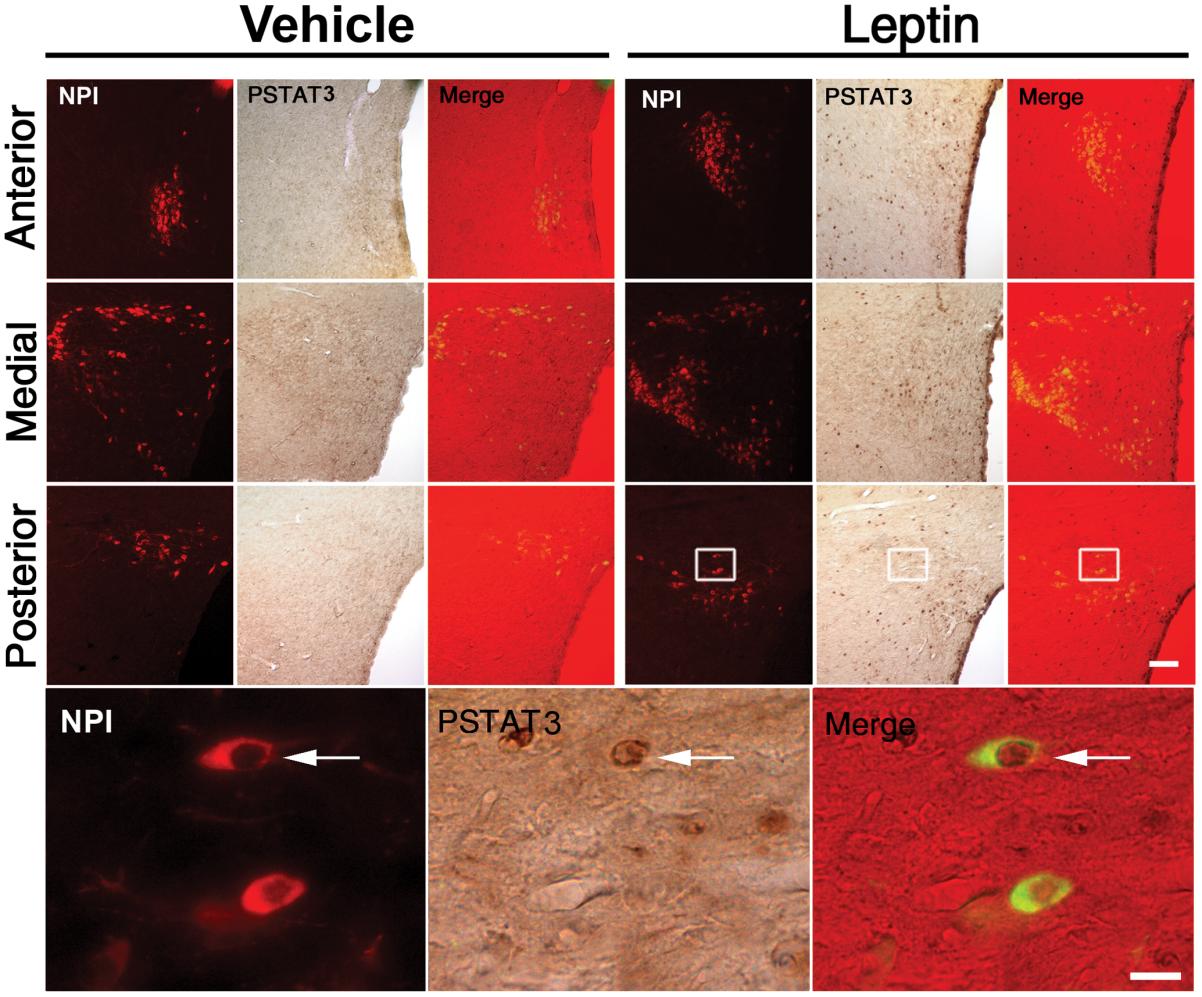
ICV leptin increased pSTAT3 in OXT neurons of the PVN in DIO rats. Left and right upper sets of panels depict low magnification photomicrographs of the anterior, medial and posterior parts of the PVN in DIO rats treated with vehicle or leptin, respectively. Red fluorescent and brown signals represent NPI-IR and pSTAT3-IR, respectively. Cells with co-localization of NPI-IR with pSTAT3-IR are seen in the merge panels. Bottom set of panels show high magnification photomicrographs of area marked in low magnification images, with arrows pointing to dual-labeled cells. Scale bars: 50 µm (Upper panels), 20 µm (Bottom panels).

We recently reported that DIO rats fed on high fat diet for 12 weeks are leptin resistant [23]: i-DIO rats are heavier and have higher plasma leptin concentrations than rats fed on regular chow; ii-ICV leptin fails to affect food intake of DIO rats; and iii-ICV leptin-induced increase of pSTAT3-IR cells in the ARC of fasted DIO rats is reduced by ∼50% as compared to control rats. In order to test if leptin activates OXT neurons in DIO rats, we performed double IHC for pSTAT3 and NPI in ICV leptin- and vehicle-treated fasted DIO animals. Total NPI-IR cells of the PVN in DIO rats were estimated in 1371±152 cells. Figure 5 depicts a set of representative images of three levels of the PVN of DIO rats subjected to vehicle or leptin treatments. We found no NPI-IR neurons positive for pSTAT3 in vehicle-treated DIO rats, while 7.1±1.8% of NPI-IR neurons of the PVN were positive for pSTAT3 in leptin-treated DIO rats (p<0.01). Of note, all NPI-IR cells positive for pSTAT3 were located in the parvocellular part of the PVN. Thus, ICV leptin is able to act on some OXT neurons of the PVN in fasted DIO rats.

**Figure 6:**
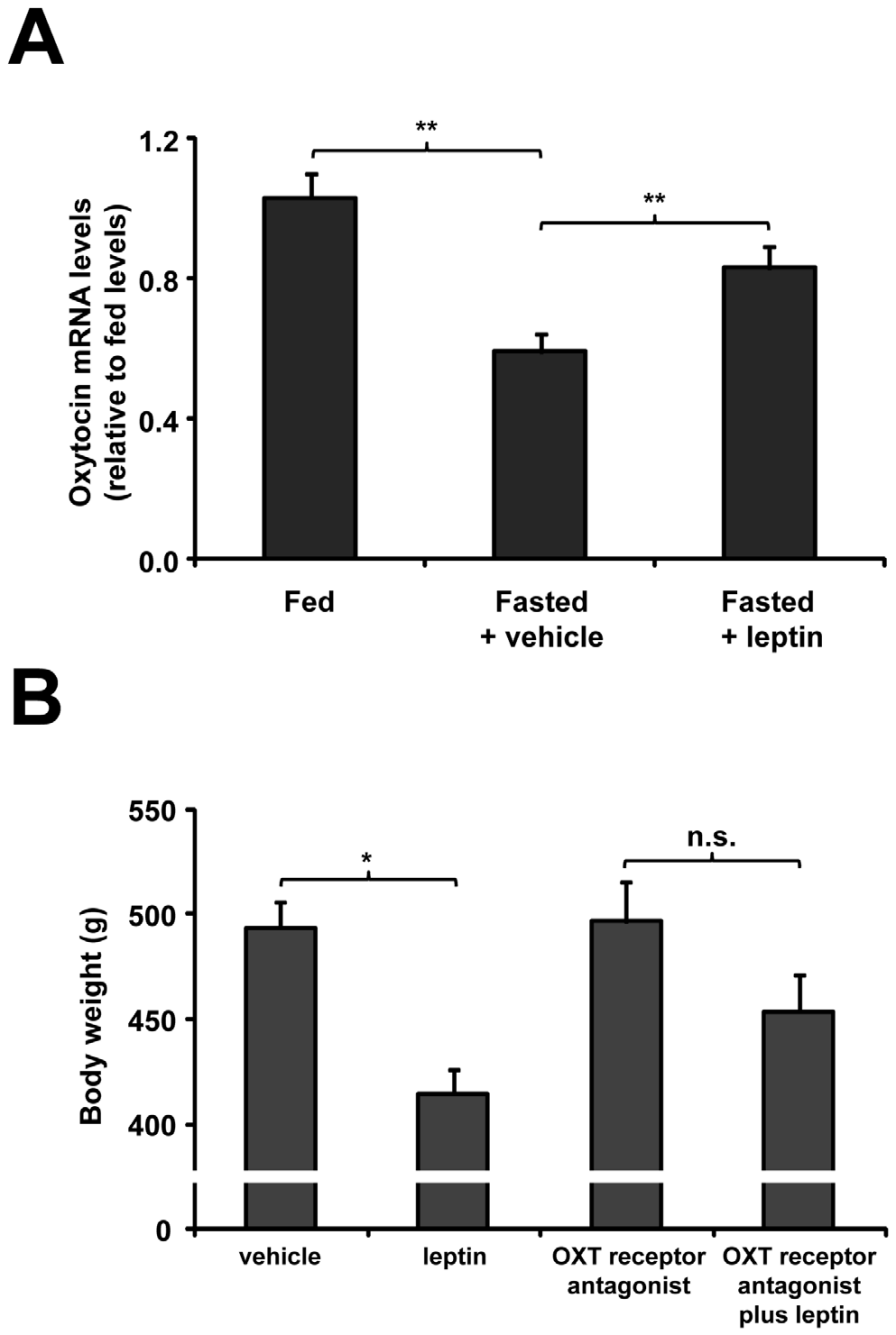
ICV leptin affected OXT gene expression in the PVN (A) and required OXT signaling to regulate body weight (B) in fasted DIO rats. **A.** OXT mRNA levels are presented as relative to levels observed in PVN of control fed rats. **B.** Body weight was determined after completion of 48 h fasting and simultaneous ICV administrations, every 6 h, of either vehicle or leptin in presence or absence of the OXT receptor antagonist. *, p<0.05. **, p<0.01. n.s., not significant.

We next tested if leptin affects OXT gene expression in the PVN of DIO rats. A*d libitum* fed DIO rats had OXT mRNA levels in the PVN similar to fed control rats (1.03±0.07 vs. 1.00±0.06 relative levels, respectively). As shown in Figure 6A, OXT mRNA levels in the PVN of DIO rats were down regulated by fasting (0.59±0.05 relative levels, p*<*0.01 vs. fed condition) and reversed by leptin treatment (0.83±0.06 relative levels, p*<*0.01 vs. vehicle-treated fasted DIO rats), in a similar fashion as observed in control rats. As shown in Figure 6B, fasted DIO rats receiving leptin had a significant reduction in body weight as compared to fasted DIO rats treated with vehicle (414±12 vs. 493±12 g, respectively, p*<*0.01). In contrast, fasted DIO rats receiving leptin and the OXT receptor antagonist failed to show any significant reduction in body weight as compared to fasted rats treated with the OXT receptor antagonist alone (454±18 vs. 497±19 g, respectively). Thus, ICV leptin regulates OXT mRNA levels in the PVN, and OXT signaling contributes to leptin-induced decrease of body weight in fasted DIO rats.

## Discussion

Here, we show that ICV leptin increases pSTAT3 in the OXT neurons of the PVN that innervate the NTS. In addition, we show that ICV leptin is able to regulate OXT mRNA levels in the PVN, and that OXT signaling may be involved in leptin-mediated regulation of body weight. This finding supports previous studies showing that OXT neurons of the PVN link the hypothalamic action of leptin to the NTS to control body weight [14], and provide further evidence suggesting that these neurons may be a direct target of leptin. We also show that ICV leptin activates OXT neurons of the PVN in obese rats, and that this action affects body weight of DIO rats in our experimental conditions.

Recent evidence has highlighted the role of OXT in the central regulation of body weight [35]. Similarly to leptin, OXT reduces body weight and fat mass through inhibition of food intake and increased energy expenditure. OXT or OXT antagonist administration reduces or increases food intake, respectively [36]–[40]. Also, OXT mRNA levels in the PVN are decreased and increased by fasting and re-feeding, respectively [10], [11]. Interestingly, OXT deficiency in the PVN mediates hyperphagia and obesity in mice with heterozygous inactivation of the transcription factor single-minded 1 [11]. Of note, OXT also increases energy expenditure since OXT or OXT receptor-deficient mice display low sympathetic tone and increased body weight and fat mass without hyperphagia [12], [13]. In addition, mice with genetic ablation of the OXT neurons are more sensitive to DIO specifically due to a energy expenditure reduction [15]. It had been previously shown that leptin inhibition of food intake is partially mediated by OXT neurons of the PVN [14]. Our current findings provide the first evidence of a link between OXT and leptin-induced increase of energy expenditure. In order to make apparent the leptin effect on energy expenditure, we ICV administrated leptin to fasted rats, in which the anorexic effect of the hormone is removed by eliminating food intake. The leptin-induced reduction in body weight observed in fasted animals occurs because leptin administration partially prevents the reduction of energy expenditure normally associated to fasting [41]. Here, we show that the further reduction in body weight observed in fasted animals ICV treated with leptin is attenuated by an OXT receptor antagonist, at a dose that had no independent effects on body weight. Thus, ICV leptin can elicit body weight changes through OXT signaling in fasted rats. However, these results should not be generalized to physiological effects of leptin on body weight regulation since they were obtained under some specific experimental conditions. Since OXT neurons of the PVN provide the largest contribution that control BAT functions [32], [42], it could be hypothesized that these neurons mediate leptin regulation of thermogenesis in BAT. OXT blockade may also cause a reduction of leptin-induced increase of other sympathetic functions and/or locomotor activity [43], [44]. Future studies will be required to test these possibilities.

Here, we show that leptin increases pSTAT3 exclusively in OXT neurons of the parvocellular PVN that innervate the NTS. The parvocellular OXT neurons give rise to the greater number of projections from the PVN to the brainstem, where they innervate several nuclei including the NTS [32], and these projections are essential for body weight regulation [45]. The NTS, which expresses high levels of OXT receptors [46], [47] and receives dense OXT innervations solely from parvocellular PVN neurons [25], [48], is known to mediate some actions of leptin on energy balance [10]. In addition, viral retrograde transynaptic studies found that the NTS participates of the neuronal circuitry regulating sympathetic outflow to BAT [49]. Thus, previous neuroanatomical studies support the possibility that innervations of OXT neurons of the PVN to NTS neurons could contribute to mediate effects on energy expenditure.

Direct leptin targets include mainly the ARC, where circulating factors has preferential access; however, leptin can also impact on other hypothalamic nuclei such as the PVN [2]–[4], [17], [50]. There is compelling evidence supporting the direct action of leptin on the rat PVN, where: i-LepR mRNA and protein are present [3], [17]; ii-peripheral and central leptin administration induces pSTAT3 [7], [8], [23], [51]; iii-leptin administration activates gene expression of suppressor-of-cytokine-signaling 3, another marker of direct leptin action [9]; iv-leptin depolarizes neurons [52]; and v-micro-injection of leptin affects some gastrointestinal functions [53]. The STAT3 phosphorylation has been extensively used as a direct measure of LepR activation since it is activated at areas that overlap well with LepR mRNA expression [3], [54], [55]. In addition, neuronal deletion of STAT3 protein or STAT3 binding site on LepR in mice reproduces the metabolic phenotype of the LepR null animal [56], [57]. Thus, our data can be interpreted as leptin directly regulates a subpopulation of OXT neurons of the PVN. In the current study, leptin was ICV-administrated in order to rule out any potential contribution of peripheral actions of the hormone. Under physiological conditions, however, the action of circulating leptin on the PVN depends also on the blood–brain barrier transport regulation and on ARC-dependent pathways, which are particularly sensitive to plasma factors. Thus, the relevance of direct leptin signaling on OXT neurons of the PVN under physiological conditions requires further studies.

Our current neuroanatomical findings raise additional and interesting considerations. When neurons belonging to the hypophyseal system were labeled (Figure 2), we observed single FG-IR cells positive of pSTAT3. These neurons, which are presumably a direct target of leptin, include likely TRH neurons since we have shown that leptin activates hypophysiotropic TRH neurons [23]. When neurons innervating the NTS were labeled (Figure 3), we found that all OXT neurons of the PVN negative for FG also lacked pSTAT3 labeling. Therefore, no other subpopulation of OXT neurons of the PVN seems to be affected by leptin. In this tracing study, we also observed single FG-IR cells positive for pSTAT3 that were negative for NPI. Thus, the PVN may contain non-OXT neurons that innervate the NTS and increase pSTAT3 in response to leptin. Interestingly, Elias *et al*. have shown that very few leptin-activated neurons of the PVN innervate sympathetic pre-ganglionic neurons in the thoracic spinal cord [34]. Thus, the NTS may be one of the main targets of the leptin-responsive neurons of the PVN. Of note, it is also plausible that leptin regulates additional OXT neurons of the PVN in a pSTAT3-independent fashion, for instance via the melanocortin system [58].

Leptin resistance is a common feature seen in obese patients that has limited the use of leptin for the treatment of obesity [1]. Studies in DIO rodents, which are widely used as a model of human obesity, have shown that the ARC is a major site of leptin resistance while some other sites within the brain remain leptin sensitive [21], [22]. These leptin-responsive brain areas in DIO rodents seem to be physiologically relevant as these animals become much less obese than *ob*/*ob* mice, which completely lack leptin signaling [22]. Over the past years, some of the specific neuronal populations that remain leptin sensitive in DIO models started to be identified. We have shown that hypophysiotropic TRH neurons of the PVN remain sensitive to leptin in DIO animals [23]. Additionally, neurons of the hypothalamic dorsomedial nucleus have been shown to remain sensitive to the direct action of leptin in DIO rodents [24]. Here we propose that parvocellular OXT neurons of the PVN could be included as another set of leptin sensitive neurons in DIO animals. According to this possibility, Wu *et al.* have shown an obligate role of OXT neurons in diet-induced energy expenditure in mice [15]. Interestingly, recent evidences indicate that OXT treatment reduces obesity, food intake and fat mass in DIO animals [59], [60]. Thus, pharmacological manipulations of OXT system may be a potential target for treating body weight-related disorders.
